# Prediction of axillary lymph node metastasis in primary breast cancer patients using a decision tree-based model

**DOI:** 10.1186/1472-6947-12-54

**Published:** 2012-06-13

**Authors:** Masahiro Takada, Masahiro Sugimoto, Yasuhiro Naito, Hyeong-Gon Moon, Wonshik Han, Dong-Young Noh, Masahide Kondo, Katsumasa Kuroi, Hironobu Sasano, Takashi Inamoto, Masaru Tomita, Masakazu Toi

**Affiliations:** 1 Department of Breast Surgery, Graduate School of Medicine, Kyoto University, Kyoto, Japan; 2 Research Fellow of the Japan Society for the Promotion of Science, Tokyo, Japan; 3 Institute for Advanced Biosciences, Keio University, Yamagata, Japan; 4 Systems Biology Program, Graduate School of Media and Governance, Keio University, Kanagawa, Japan; 5 Medical Innovation Center, Kyoto University Graduate School of Medicine, Kyoto, Japan; 6 Department of Environment and Information Studies, Keio University, Kanagawa, Japan; 7 Department of Surgery, Seoul National University College of Medicine, Seoul, Republic of Korea; 8 Department of Health Care Policy and Management, Graduate School of Comprehensive Human Sciences, University of Tsukuba, Ibaraki, Japan; 9 Department of Surgery, Tokyo Metropolitan Cancer and Infectious Diseases Center, Komagome Hospital, Tokyo, Japan; 10 Department of Pathology, Tohoku University Hospital and School of Medicine, Miyagi, Japan; 11 Department of Breast Surgery, Tenri Hospital, Nara, Japan

**Keywords:** Breast cancer, Lymph node metastasis, Data mining, Alternating decision tree

## Abstract

**Background:**

The aim of this study was to develop a new data-mining model to predict axillary lymph node (AxLN) metastasis in primary breast cancer. To achieve this, we used a decision tree-based prediction method—the alternating decision tree (ADTree).

**Methods:**

Clinical datasets for primary breast cancer patients who underwent sentinel lymph node biopsy or AxLN dissection without prior treatment were collected from three institutes (institute A, *n* = 148; institute B, *n* = 143; institute C, *n* = 174) and were used for variable selection, model training and external validation, respectively. The models were evaluated using area under the receiver operating characteristics (ROC) curve analysis to discriminate node-positive patients from node-negative patients.

**Results:**

The ADTree model selected 15 of 24 clinicopathological variables in the variable selection dataset. The resulting area under the ROC curve values were 0.770 [95% confidence interval (CI), 0.689–0.850] for the model training dataset and 0.772 (95% CI: 0.689–0.856) for the validation dataset, demonstrating high accuracy and generalization ability of the model. The bootstrap value of the validation dataset was 0.768 (95% CI: 0.763–0.774).

**Conclusions:**

Our prediction model showed high accuracy for predicting nodal metastasis in patients with breast cancer using commonly recorded clinical variables. Therefore, our model might help oncologists in the decision-making process for primary breast cancer patients before starting treatment.

## Background

Axillary lymph node (AxLN) metastasis is one of the most important prognostic factors in patients with primary breast cancer for predicting survival [1-4]. Sentinel lymph node (SLN) biopsy is widely used to determine AxLN status and avoids AxLN dissection (ALND). However, SLN biopsy is an invasive procedure. Therefore, predicting AxLN metastasis before SLN biopsy using commonly recorded clinical variables would be helpful for oncologists and could avoid this procedure, especially in elderly patients or patients with complications. Consequently, many mathematical models have been developed to predict AxLN metastasis, including nomograms and scoring systems [5-14]. For example, the Memorial Sloan-Kettering Cancer Center (MSKCC) developed a nomogram to predict the presence of SLN metastasis [6] that is now used worldwide.

Technically, nomograms use multiple logistic regression (MLR) to predict a binary outcome based on a combination of risk factors. This well-established method has a limitation in that it incorporates only a few independent variables so that the model can accurately predict risk in independent datasets, by avoiding over-fitting to the given datasets. Such prediction models should also tolerate missing values, which are common in clinical datasets. Thus, new methods to cope with a greater number of variables and that provide accurate prediction and robustness against missing values are required.

Machine learning has been applied to problems across many fields, including bioinformatics [15], and it is thought to overcome or reduce the impact of the limitations of MLR. Here, we used the alternating decision tree (ADTree) [16,17] as a core algorithm. This algorithm consists of a root node and multiple simple decision trees in which an index is associated with each leaf node, and its final predictive value is the sum of the indices of the leaf nodes fulfilling the patients’ condition. This algorithm also differs from standard ‘if–then’ decision trees and classification and regression trees (CART). The ADTree method has several advantages compared with these other machine learning algorithms, including: (1) several comparative studies have shown higher accuracy and versatility for ADTree than other machine learning methods [18,19]; and (2) the ADTree model structure is less complex than other methods [16], which facilitates model interpretation and reduces the need for model optimization.

The purpose of this study was to develop a new mathematical model to predict AxLN metastasis in patients with primary breast cancer using preoperative clinico-pathological information.

## Methods

### Patients

The training datasets consisted of consecutive patients who were treated at two institutions in Japan. Patients with histologically confirmed primary invasive breast cancer who underwent SLN biopsy or ALND without prior treatment were eligible for this study. We included patients whose maximum tumor size was ≤ 4 cm. We identified 148 patients from the Tokyo Metropolitan Cancer and Infectious Diseases Centre Komagome Hospital who were treated between 2005 and 2006 (Tokyo dataset) and 143 patients from Kyoto University Hospital treated between 2008 and 2009 (Kyoto dataset).

The external validation dataset was collected from Seoul National University Hospital, Korea, and consisted of patients consecutively treated between January 6, 2010, and April 16, 2010 (Seoul dataset). We included 174 patients who underwent SLN biopsy and met the same eligibility criteria as the modeling dataset. All datasets were collected after establishing the methodology for SLN biopsy, and no significant difference in SLN biopsy accuracies was expected [20,21].

The study protocol was approved by the institutional review board at Kyoto University Hospital. All patient data were anonymized and allocated numbers according to Japanese ethical guidelines for epidemiologic research.

### Data collection and sentinel lymph node biopsy

Clinical data collected included age, body mass index (BMI), menopausal status, physical findings (based on inspection or palpation), diagnostic mammography and ultrasonography findings, pathological findings from needle biopsy before treatment (*e.g.*, histological type, histological/nuclear grade, estrogen receptor status, progesterone receptor status, and human epidermal growth factor receptor 2 [HER2] status), and type of axillary surgical procedure (SLN biopsy or ALND) as predictive variables. Pathological findings from surgical specimens (presence or absence of lymph node metastasis) were used as outcome variables for prediction by the ADTree model. All data were retrospectively collected from databases maintained at each institution.

The grading criteria were established by a committee of specialists from the fields of breast surgery, diagnostic radiology and pathology. We reviewed all of the images from which mammographic and ultrasonographic variables were obtained, and these parameters were determined using Japanese diagnostic guidelines for mammography and ultrasonography based on the American College of Radiology Breast Imaging Reporting and Data System [22]. These variables were reviewed by physicians certified for imaging diagnosis by the relevant accreditation organizations in Japan.

The techniques used for SLN biopsy and histological evaluations are described elsewhere [21]. In the Tokyo dataset, SLNs were identified using a radioactive tracer (^99m^Tc-phytate). In the Kyoto dataset, they were identified using blue dye and a fluorescence navigation technique using indocyanine green. In the Seoul dataset, SLNs were identified using both blue dye and a radioactive tracer. At each institution, the SLNs were step-sectioned, stained with hematoxylin and eosin (H&E), and diagnosed by trained pathologists. Lymph nodes obtained after ALND were evaluated using a single H&E-stained section from each node. Metastases were defined as the presence of a tumor deposit > 0.2 mm in diameter in at least one lymph node. Several clinical trials have reported no significant differences in the identification rate or accuracy of SLN methodologies [20,23,24].

### Data analysis

A summary of the model development and validation procedure is shown in Appendix A (Additional file 1). The model development phase consisted of three steps. First, bias-control virtual datasets were generated from the Tokyo dataset by randomly selecting individuals allowing for redundant selection. These datasets contained an approximately equal ratio of patients negative and positive for AxLN. Second, a prediction model containing multiple ADTrees was trained on a generated dataset, and the mean value of the individual trees’ predictions values was used to enhance the accuracy and generalization ability in a process referred to as the ensemble technique [25]. This model development procedure was repeated for different modeling conditions, *e.g.* the number of nodes, and all virtual datasets. Third, we selected the model yielding the best area under the receiver operating characteristics (ROC) curves (AUC) value with the Kyoto dataset. Finally, we performed external validation of the chosen model using the Seoul dataset.

The established model was further evaluated as follows. First, we performed bootstrap analysis using the Seoul dataset to obtain unbiased estimates of the developed model. Second, the relative importance of the variables in the model was analyzed by randomly changing the values of each variable (sensitivity analysis). Third, missing values in the Seoul datasets were changed to random values to evaluate the model’s tolerance against missing values (missing value analysis). Fourth, the number of trees in the prediction model was reduced to evaluate the relationship between the number of variables in the model and the prediction accuracy (pruning analysis).

Two hundred bias-controlled datasets were generated using different random values. The number of nodes (called boosting iterations) in an ADTree was expanded from 10, 11, … to 20 in each trial. For the ensemble procedure, we randomly sampled individuals to generate multiple datasets, and the averaged prediction of the trained models for each dataset was used [26]. In this ensemble procedure, the number of ADTrees ranged from 2, 3, …, to 20, with a random seed to generate random values (1, 2, …, and 10). Two hundred replicates with different random values were generated for each bootstrap, sensitivity and missing value analysis.

Weka (ver. 3.6.1; University of Waikato, Hamilton, NZ) [27] was used for resampling, the ensemble procedure and ADTree development. The Mann–Whitney test and AUCs with 95% confidence interval (CI) were calculated using GraphPad Prism version 5.04 (GraphPad Software, Inc., San Diego, CA). JMP® (ver. 7.0.1, SAS Institute, Cary, NC, USA) was used for other statistical analyses.

## Results

The clinicopathological characteristics of patients in each dataset are summarized in Table 1. The proportion of patients with AxLN metastasis was 29.7%, 30.8% and 23.6% in the Tokyo, Kyoto and Seoul datasets. The proportion of patients with AxLN metastasis in the Seoul dataset was not significantly different from the other datasets (*P* = 0.292).

**Table 1:** Patient characteristics and incidence of lymph node metastasis

**Variables**	**Tokyo dataset**		**Kyoto dataset**		**Seoul dataset**		***P*-value**^**§**^
	**No**	**%**	**No**	**%**	**No**	**%**	
No. of patients	148	(100)	143	(100)	174	(100)	
Age							<0.001
Median	55		60		50		
Range	(31–85)		(26–88)		(25–74)		
Body mass index							0.019
Median	22.9		22.3		23.2		
Range	(16.6–43.2)		(14.8–31.4)		(17.8–37)		
Unknown	3	(2)	0	(0)	1	(0.6)	
Clinical T classification							0.2621
T1	102	(68.9)	100	(69.9)	108	(62.1)	
T2	46	(31.1)	43	(30.1)	66	(37.9)	
Clinical N classification							0.002
N0	137	(92.6)	135	(94.4)	174	(100)	
N1	11	(7.4)	8	(5.6)	0	(0)	
Skin dimpling							<0.001
Yes	22	(14.9)	14	(9.8)	2	(1.1)	
No	109	(73.6)	129	(90.2)	172	(98.9)	
Unknown	17	(11.5)	0	(0)	0	(0)	
Nipple discharge							0.238
Yes	6	(4.1)	2	(1.4)	3	(1.7)	
No	138	(93.2)	141	(98.6)	170	(97.7)	
Unknown	4	(2.7)	0	(0)	1	(0.6)	
Mammography							
Presence of masses							0.284
Yes	90	(60.8)	88	(61.5)	102	(58.6)	
Focal asymmetry	22	(14.9)	20	(14)	39	(22.4)	
No	35	(23.6)	26	(18.2)	33	(19)	
Unknown	1	(0.7)	9	(6.3)	0	(0)	
Presence of calcifications							0.037
Yes	67	(45.3)	44	(30.8)	59	(33.9)	
No	81	(54.7)	94	(65.7)	115	(66.1)	
Unknown	0	(0)	5	(3.5)	0	(0)	
Shape of calcifications							0.010
Fine branching or casting	4	(6)	1	(2.3)	3	(5.1)	
Pleomorphic	9	(13.4)	11	(25)	21	(35.6)	
Amorphous or indistinct	43	(64.2)	27	(61.4)	35	(59.3)	
Round or benign	11	(16.4)	4	(9.1)	0	(0)	
Unknown	0	(0)	1	(2.3)	0	(0)	
Distribution of calcifications							0.024
Linear or segmented	26	(38.8)	14	(31.8)	22	(37.3)	
Grouped or clustered	30	(44.8)	29	(65.9)	36	(61)	
Regional or diffuse	9	(13.4)	1	(2.3)	1	(1.7)	
Unknown	2	(3)	0	(0)	0	(0)	
Ultrasonography							
Presence of masses							0.264
Yes	142	(95.9)	133	(93)	161	(92.5)	
No	5	(3.4)	10	(7)	13	(7.5)	
Unknown	1	(0.7)	0	(0)	0	(0)	
Multifocality							0.114
Yes	27	(19)	14	(10.5)	21	(13)	
No	115	(81)	119	(89.5)	140	(87)	
Maximum tumor size (mm)							0.004
Median	16		16.1		19		
Range	(4–37)		(5–35)		(4–37)		
Depth/width ratio							0.001
Median	0.72		0.67		0.64		
Range	(0.31–1.36)		(0.22–1.43)		(0.33–1.27)		
Unknown	0	(0)	9	(6.8)	0	(0)	
Echogenic halo							<0.001
Yes	32	(22.5)	62	(46.6)	38	(23.6)	
No	109	(76.8)	71	(53.4)	123	(76.4)	
Unknown	1	(0.7)	0	(0)	0	(0)	
Interruption of the anterior border of the mammary gland							0.807
Yes	99	(69.7)	91	(68.4)	106	(65.8)	
No	43	(30.3)	42	(31.6)	54	(33.5)	
Unknown	0	(0)	0	(0)	1	(0.6)	
Detection of LNs							0.130
Detectable	49	(33.1)	37	(25.9)	56	(32.2)	
Not detectable	82	(55.4)	105	(73.4)	117	(67.2)	
Unknown	17	(11.5)	1	(0.7)	1	(0.6)	
Maximum size (mm) of LNs							0.010
Median	11		10		10		
Range	(5–22)		(3–32)		(4–17)		
Unknown	0	(0)	4	(10.8)	1	(1.8)	
Hilum of LNs							0.021
Detectable	43	(87.8)	27	(73)	36	(64.3)	
Not detectable	6	(12.2)	9	(24.3)	20	(35.7)	
Unknown	0	(0)	1	(2.7)	0	(0)	
Histological type							0.584
Invasive ductal carcinoma	135	(91.2)	129	(90.2)	160	(92)	
Invasive lobular carcinoma	5	(3.4)	3	(2.1)	7	(4)	
Other specific types	8	(5.4)	11	(7.7)	7	(4)	
Estrogen receptor^†^							0.023
Positive	119	(80.4)	114	(79.7)	121	(69.5)	
Negative	27	(18.2)	29	(20.3)	53	(30.5)	
Unknown	2	(1.4)	0	(0)	0	(0)	
Progesterone receptor^†^							0.427
Positive	83	(56.1)	89	(62.2)	96	(55.2)	
Negative	63	(42.6)	54	(37.8)	78	(44.8)	
Unknown	2	(1.4)	0	(0)	0	(0)	
HER2^‡^							0.019
Positive	18	(12.2)	11	(7.7)	29	(16.7)	
Negative	121	(81.8)	131	(91.6)	125	(71.8)	
Unknown	9	(6.1)	1	(0.7)	20	(11.5)	
Histological/nuclear grade							<0.001
1	64	(43.2)	43	(30.1)	4	(2.3)	
2	47	(31.8)	63	(44.1)	82	(47.1)	
3	27	(18.2)	36	(25.2)	88	(50.6)	
Unknown	10	(6.8)	1	(0.7)	0	(0)	
LN metastasis							0.292
Yes	44	(29.7)	44	(30.8)	41	(23.6)	
No	104	(70.3)	99	(69.2)	133	(76.4)	

Note:

Abbreviations: LN, lymph node.

^†^Estrogen receptor or progesterone receptor positive was defined as ≥10% positively stained cells on immunohistochemical (IHC) testing.

^‡^HER2 positive was defined as IHC 3+ or positive on fluorescence *in situ* hybridization testing.

^§^The *χ*^2^ test or Kruskal–Wallis test was used depending on the distribution of patients in each variable and dataset.

**Figure 1:**
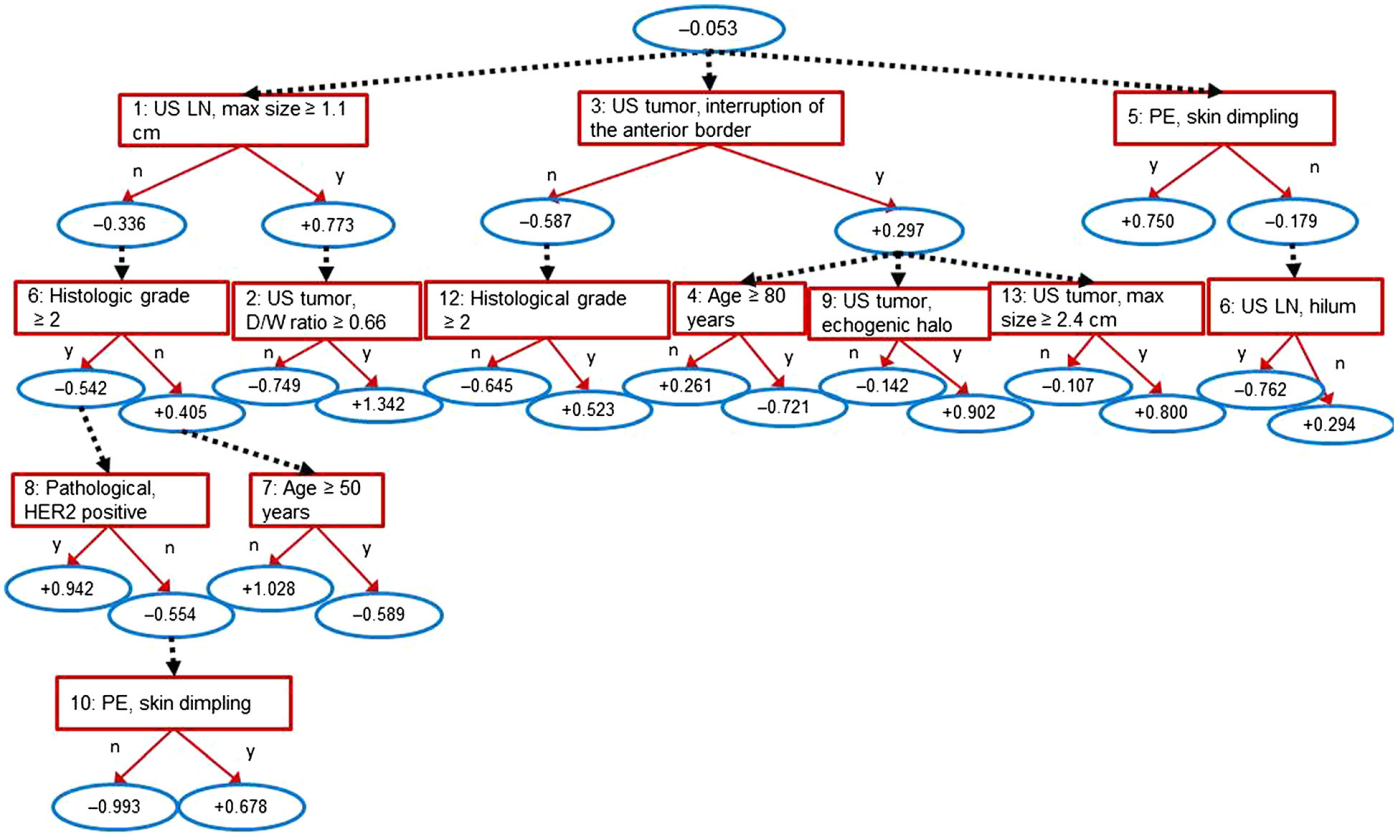
**ADTree model.** The final prediction model consisted of five ADTree-based prediction models; the other four models are depicted in Appendix B (Additional file 1). The method used to calculate the prediction score for each model is shown in Appendix C (Additional file 1). The final prediction was calculated by calculating the mean score of the five ADTree models.

The model with the best AUC value in the Kyoto dataset included five ADTrees with 13 nodes (Figure 1 and Appendix B (Additional file 1)). A total of 15 variables were included: age, BMI, seven ultrasonographic variables (maximum tumor size, tumor depth/width ratio, multifocality, echogenic halo, interruption of the anterior border of the mammary gland, maximum size of lymph nodes, and a loss of hilum in lymph nodes), two mammographic variables (shape and distribution of calcification), two physical examination variables (skin dimpling and nipple discharge) and two pathological variables (histological/nuclear grade, HER2 status). The method used to calculate the score is shown in Appendix C (Additional file 1).

**Figure 2:**
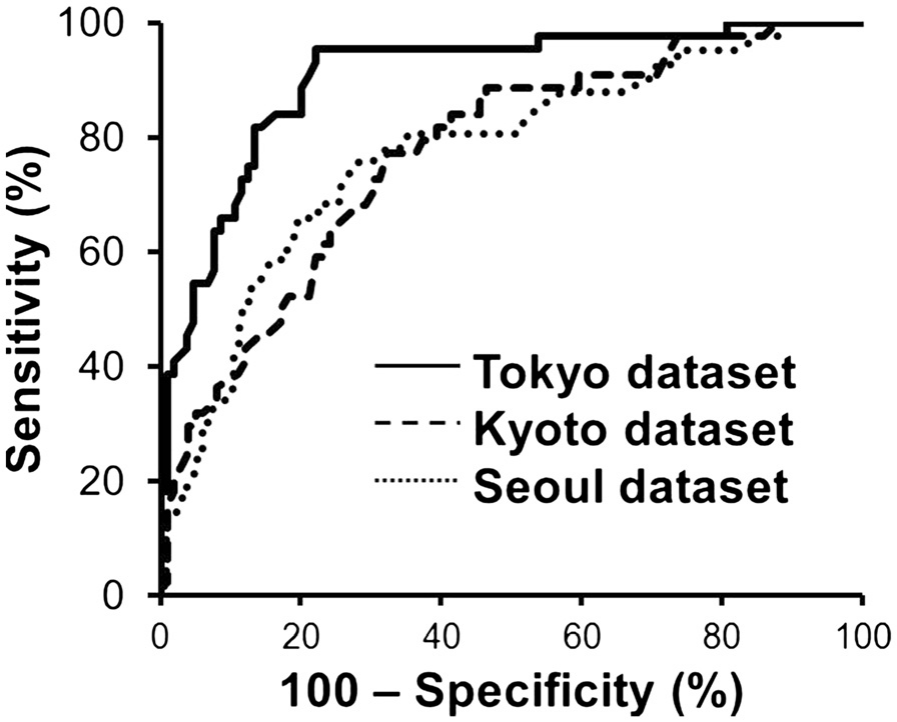
**Receiver operating characteristic (ROC) curves of the prediction model.** The area under the ROC curve (AUC) values were 0.917 (95% CI: 0.871–0.964, *P* < 0.0001), 0.770 (95% CI: 0.689–0.850, *P* < 0.0001) and 0.772 (95% CI: 0.689–0.856, *P* < 0.0001) for the Tokyo, Kyoto and Seoul (validation dataset) datasets, respectively.

**Figure 3:**
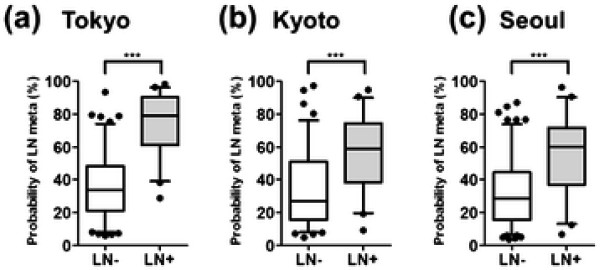
**Box plots showing the predicted probabilities of lymph node metastasis for the Tokyo (a), Kyoto (b) and Seoul (c) datasets.** In each figure, the boxes show the actual number of lymph node-negative (LN–) and -positive (LN+) patients, respectively. The whisker box-plots indicate the 5^th^, 25^th^, 50^th^, 75^th^ and 95^th^ percentiles (from the bottom bar to the upper bar) of the predicted probabilities. The probabilities <5% and >95% are plotted individually. The differences between LN– and LN + were statistically significant (*P* < 0.0001; Mann–Whitney test) in all datasets. The median predicted probabilities of LN– and LN + were **(a)** 33.5 (95% CI: 31.8–39.4) and 78.9 (95% CI: 69.3–80.4), **(b)** 33.6 (95% CI: 29.1–38.0) and 58.9 (95% CI: 49.3–62.9), and **(c)** 32.3 (95% CI: 28.8–35.8) and 59.9 (95% CI: 48.2–62.6).

The ROC curves for each dataset are shown in Figure 2. The AUC values were 0.917 (95% CI: 0.871–0.964, *P* < 0.0001) for the Tokyo dataset, 0.770 (95% CI: 0.689–0.850, *P* < 0.0001) for the Kyoto dataset and 0.772 (95% CI: 0.689–0.856, *P* < 0.0001) for the Seoul dataset. Box plots of the predicted probabilities of AxLN metastasis are shown in Figure 3. The model discriminated node-positive patients from node-negative patients at statistically significant levels (*P* < 0.0001), although there was some overlap of the predicted probability distribution of node-negative and node-positive status in each dataset.

The mean AUC values yielded by bootstrap analysis remained high for each dataset, being 0.916 (95% CI: 0.913–0.919), 0.766 (95% CI: 0.760–0.772) and 0.768 (95% CI: 0.763–0.774) for the Tokyo, Kyoto and Seoul datasets, respectively. A calibration plot of the model developed using the Kyoto and Seoul datasets is shown in Appendix D (Additional file 1). The predicted probabilities were divided into quintiles according to their values, and the mean and actual frequencies of AxLN metastasis were plotted for each quintile.

**Figure 4:**
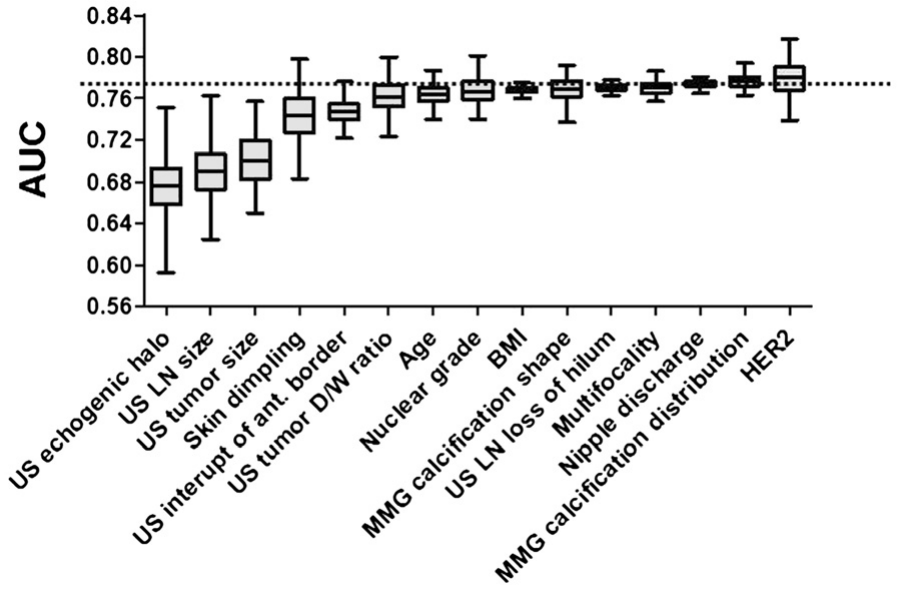
**Sensitivity analysis using the Seoul dataset.** Whisker-box plots showing 0, 25, 50, 75 and 100% (from the bottom bar to the upper bar) of the area under the curve (AUC) values when the variable was randomly replaced 200 times. The horizontal dashed line indicates the AUC value in the external validation test without any variable replacement.

In the sensitivity analysis, the AUC values decreased remarkably when the following variables were randomly replaced: echogenic halo, maximum size of the lymph nodes, maximum size of the tumor, skin dimpling, and interruption of the anterior border of the mammy gland. This indicates that the developed model was more sensitive to this variable than the other variables, which hardly affected AUC values (Figure 4). In the missing value analysis, 33 and 19 patients with missing values were selected from the Kyoto and Seoul datasets, and we validated the developed model by replacing missing values with random values. This procedure was repeated 200 times for each dataset, and the mean AUC values were 0.884 (95% CI: 0.882–0.887) and 0.688 (95% CI: 0.684–0.692) for the Kyoto and Seoul datasets, respectively. In the pruning analysis, the number of trees was reduced from 5 to 1, and AUC values were calculated for the Tokyo datasets in cross-validation mode, in addition to the Kyoto and Seoul datasets (Appendix E (Additional file 1)).

The predictive performance of the MSKCC nomogram and a scoring system developed at Russells Hall Hospital, United Kingdom, were evaluated using the Seoul dataset [6,28]. Both models included lymphovascular invasion (LVI) as an input variable. However, LVI is not routinely reported for needle biopsy samples because of its uncertain diagnostic role [29]. As preoperative pathological diagnosis in the Seoul dataset was performed by needle biopsy, we used LVI status assessed on surgical specimens. The resulting AUC values were 0.664 (95% CI; 0.560–0.768, *P* = 0.0033) for the nomogram and 0.620 (95% CI; 0.509–0.731, *P* = 0.0032) for the scoring system using individuals without missing values (*n* = 131) (Appendix F (Additional file 1)). The AUC value using the corresponding patients in the Seoul dataset was 0.777 (95% CI: 0.689–0.864, *P* < 0.001) for ADTree.

## Discussion

A data-mining model generated using the ADTree ensemble technique improved the prediction of AxLN metastasis in patients with primary breast cancer, compared with older models such as the MSKCC nomogram. Evaluation using an external validation dataset and bootstrap analysis revealed high AUC values of 0.772 and 0.768, respectively. However, the prediction was not perfect and there are several issues that may affect the prediction performance.

Different variations in patient variables between the training and validation datasets possibly lowered the AUC values for the external validation. There were fewer patients with AxLN metastasis in the Seoul dataset (23.6%) compared with the Tokyo (29.7%) and Kyoto (30.8%) datasets, although this was not statistically significant (*P* = 0.29) (Table 1). One reason for this difference is that patients who underwent ALND were included in the Tokyo and Kyoto datasets (14.8%) but not in the Seoul dataset. Interestingly, the number of node-positive patients in the Tokyo and Kyoto datasets was slightly higher among patients who underwent ALND compared with those who underwent SLN (39% *vs.* 29%), although this was not significant (*P* = 0.15). Despite these differences, the AUC values for the Kyoto and Seoul datasets were similar (0.770 and 0.772, respectively).

The calibration plot (Appendix D (Additional file 1)) revealed that the predictive probability for the AxLN metastasis high-risk group was overestimated in both the Kyoto and Seoul datasets. Controlled bias in the training dataset consisting of approximately 50% of AxLN-positive patients (Appendix A (Additional file 1)) likely introduced this overestimation. As demonstrated by Rouzier *et al.*[30], the calibration curves for the Seoul dataset were improved (corrected) by fitting the data to the Kyoto dataset using a polynominal function, which resulted in near-ideal lines (*i.e.y* = *x*). Meanwhile, the calibration plots for the lower risk groups were relatively good, even without correction, for both the Kyoto and Seoul datasets.

Sensitivity analysis revealed the degree of influence of the variables in the developed model (Figure 1 and Appendix B (Additional file 1)). In this analysis, the values of each variable were randomized (Figure 4). Of the variables causing a greater decrease in AUC values, AxLN size is directly associated with lymph node metastasis. Tumor size is used as a predictive factor in the MSKCC nomogram [6]. Echogenic halo, interruption of the anterior border the mammary gland on ultrasonography, and skin dimpling are features that reflect tumor infiltration into the surrounding tissue [31,32]. Therefore, these variables might represent tumor characteristics in the prediction models.

The mean AUC values obtained for the missing value analysis (0.884 for Kyoto and 0.688 for Seoul) were very different from those obtained for all individuals (0.770 for Kyoto and 0.772 for Seoul) because of the small number of individuals with missing values. However, the differences between the upper and lower CIs were small (0.0047 for Kyoto and 0.0081 for Seoul), which indicates that the developed model has low sensitivity to missing values. One possible reason for this feature is that ADTree can calculate a range of predictive probabilities, even for cases with missing values (see the legend of Appendix C (Additional file 1)). By contrast, standard ‘if–then’ decision trees and CART models cannot calculate this probability. In addition to the simple structure and high accuracy of ADTree analysis, this tolerance to the missing value is also valuable when applying machine learning to clinical data with missing values.

In the pruning analysis, the AUC values for the datasets from all three institutes generally improved according to the number of ADTrees in the prediction model (Appendix E (Additional file 1)). Although increasing the number of trees resulted in a more complex model that requires more calculation time for prediction, the model developed using the ensemble procedure showed improved accuracy and generalizability.

The AUC value of the MSKCC nomogram for the authors’ own external validation sets was 0.754 [6], which is similar to our own for the Seoul dataset (0.772). Therefore, the AUC values of the developed model, the MSKCC nomogram, and the Russells Hall Hospital scoring system were compared with an external validation dataset (Seoul), which yielded values of 0.777 (95% CI: 0.689–0.864, *P* < 0.001), 0.664 (95% CI: 0.560–0.768, *P* = 0.0033) and 0.620 (95% CI: 0.509–0.731, *P* = 0.0032), respectively (Appendix F (Additional file 1)). The higher AUC value for our ADTree method might be attributed to the flexible model structure and the greater number of variables incorporated into the model. By comparison, the main advantage of both the MSKCC nomogram and the Russells Hall Hospital scoring system is that they require a small number of variables, which can facilitate data collection and interpretation of the model. Thus, these features of each modeling method represent trade-offs that should be considered when applying the models.

In addition to AUC value-based prediction performance, the false-negative rate (FNR) of the prediction model is also important when applying these models in clinical settings. For example, when a predictive value of ≤ 20% is defined as low risk for AxLN metastasis, the FNR of both the ADTree model and the MSKCC nomogram using the Seoul dataset was relatively good (5.3% and 2.6%, respectively). However, the nomogram predicted that only 6.9% of the patients were AxLN negative, compared with 23.7% using the developed model.

Unlike the MSKCC nomogram and our ADTree model, Reyal et al. developed MLR-based nomograms using the molecular subtype classification defined by a combination of ER and HER2 status with clinical parameters that included tumor size, LVI and age [33]. The decision to use ER/HER2 subtype might be attributed to the expected relationship between intrinsic breast cancer subtype and lymph node metastasis. Instead, we treated these variables as independent possible predictive factors and ADTree did not select ER status, but did select HER2 status in model development. Interestingly, HER2 status showed the lowest sensitivity in our model and the contribution of this subtype-related variable to AxLN metastasis was not significant in our study.

There are several limitations and perspectives to be discussed. First, to eliminate inter-institute or inter-interpreter variations, a standardized ultrasonography/mammography scoring system is vital because these variables are key factors for the accurate prediction of AxLN metastasis. Since a larger number of variables is required to achieve accurate prediction, unlike conventional prediction models or scoring systems, a web-based user interface, such as the one used for the MSKCC nomogram [6], will help to encourage its use and to ensure it is used correctly. In addition to calculating the probability of AxLN metastasis, a web-based platform can also assist with data collection and ensure the prediction model is kept up to date. Alternatively, machine learning-based medical classification systems have been developed following the introduction of electronic medical record systems [34-36]. Integrating prediction tools with electronic record systems will enable researchers not only to improve classification algorithms using high-dimensional datasets, but also to avoid time and effort transferring data into the classification system. Although the variables used in our developed model are frequently assessed in preoperative examinations, our proposed model is very flexible as it can incorporate new diagnostic methods or criteria. We are now developing a web-based platform to allow wider use of our model. Finally, further validation using prospective and larger datasets is indispensable before it can be used clinically.

## Conclusions

In summary, we have developed a new data-mining approach based on a combination of ADTrees to predict AxLN status in patients with primary breast cancer, as a case study. The modeling method showed accurate and versatile prediction using datasets from three institutions, despite using a large number of variables. This is one of the main benefits of using data-mining methods, unlike conventional MLR methods that can only use a few independent variables to eliminate multicollinearity. The robustness of the model against missing values is also an important property of prediction models. We believe that the approach used here could replace the conventional statistical methods and provide useful information to aid decision-making before starting treatment.

## Abbreviations

AxLN, Axillary lymph node; ADTree, Alternating decision tree; ROC, Receiver operating characteristics; AUC, Area under the receiver operating characteristics curve; SLN, Sentinel lymph node; ALND, Axillary lymph node dissection; MLR, Multiple logistic regression; LVI, Lymphovascular invasion; FNR, False-negative rate; MSKCC, Memorial sloan-kettering cancer center; BMI, Body mass index; HER2, Human epidermal growth factor receptor 2; CI, Confidence interval.

## Competing interests

The authors declare that they have no competing interests.

## Authors' contributions

MT (Takada) carried out the statistical analysis. MS performed data-mining analysis. MT, MS and YN drafted the manuscript. HM, WH and DN collected the validation data and drafted the manuscript. MK helped to design the study and helped to draft the manuscript. KK collected the training data. HS, TI and MT (Tomita) helped to design the study. MT (Toi) conceived the fundamental idea, designed the study and drafted the manuscript. All authors read and approved the final manuscript.

## Pre-publication history

The pre-publication history for this paper can be accessed here:

http://www.biomedcentral.com/1472-6947/12/54/prepub

## Supplementary Material

Additional file 1 Appendix A: Processes used to develop the predictive model. Additional B: ADTree-based prediction models. Additional C: Calculation of the predictive score in each ADTree model. Additional D: Calibration plots of the ADTree-based model for the Kyoto and Seoul datasets. Additional E: AUC values and the number of nodes in the pruning analysis. Additional F: ROC curves of the ADTree model, the MSKCC nomogram and the Russells Hall Hospital scoring system using the Seoul dataset (*n* = 131). Click here for file
