# Supplementary material for: Prediction of axillary lymph node metastasis in primary breast cancer patients using a decision tree-based model
Source: BMC Med Inform Decis Mak. 2012 Jun 13;12:54. doi: 10.1186/1472-6947-12-54 (PMC3407483; doi:10.1186/1472-6947-12-54)
Supplement: Additional file 1 — Appendix A: Processes used to develop the predictive model. Additional B: ADTree-based prediction models. Additional C: Calculation of the predictive score in each ADTree model. Additional D: Calibration plots of the ADTree-based model for the Kyoto and Seoul datasets. Additional E: AUC values and the number of nodes in the pruning analysis. Additional F: ROC curves of the ADTree model, the MSKCC nomogram and the Russells Hall Hospital scoring system using the Seoul dataset (n = 131). [file 1472-6947-12-54-S1.doc]

**Additional File 1**

**Appendix A** Processes used to develop the predictive model. **(a)** In the model development phase, bias-controlled datasets were generated from the Tokyo dataset by randomly selecting individuals, allowing redundant selection. Each generated dataset contained an approximately equal ratio of AxLN-negative and -positive patients. ADTree models were then developed using the generated datasets under different conditions, such as the number of nodes in an ADTree and the number of ADTrees in a prediction model. The model yielding the best area under the receiver operating characteristics (ROC) curve (AUC) value using the Kyoto dataset was selected. (**b)** The Seoul dataset, which was not used in the development phase, was used to evaluate the selected model.

**Appendix B** ADTree-based prediction models. One of the ADTree-based prediction models is shown in Figure 1. The final prediction score represents the mean score of the five ADTree models. The probability of lymph node metastasis (%) is determined using the formula (score_pred_ – score_min_) / (score_max_ – score_min_) × 100, where score_pred_, score_min_, and score_max_ are the predicted and theoretical minimum and maximum final scores, respectively.

**Appendix C** Calculation of the predictive score in each ADTree model. An individual tree’s score is determined by taking the value of the top node and adding those of the children nodes. When child nodes are linked to a parent node by a dashed line, the scores of all child nodes are included in the calculation. When child nodes are linked to a parent node by a solid line, only the score of the child node that fulfills the branching condition is included in the calculation. The nodes in red were involved in the calculation when a patient had the following features: A, No; B, No; C, 1; D, Z; E, Yes; and F, 5. When a node has missing value, the range of the predictive score can be calculated by considering the both branching child nodes; e.g. when F is missing, the range is from -0.2 to 0.6.

**Appendix D** Calibration plots of the ADTree-based model for the Kyoto **(a)** and Seoul **(b)** datasets. The predicted value for each variable was divided into quintiles according to the predictive probability, and the mean predictive probability and the actual frequency of lymph node metastasis were plotted for each quintile (triangles). A polynominal formula for calibration correction was developed using the Kyoto dataset and applied to the Seoul dataset. Circular dots show the corrected prediction.

**Appendix E** AUC values and the number of nodes in the pruning analysis The X-axis shows the number of ADTrees in the prediction model. Black, white and gray bars represent the AUC values of the Tokyo (cross validation), Kyoto and Seoul datasets, respectively. The line shows the number of nodes in the prediction model. For cross validation, the Tokyo dataset was randomly split into a 9:1 ratio. A model was developed using the larger dataset and evaluated using the smaller dataset. This process was repeated 10 times and the AUC was calculated based on evaluated data.

**Appendix F** ROC curves of the ADTree model, the MSKCC nomogram and the Russells Hall Hospital scoring system using the Seoul dataset (*n* = 131). The AUC values were 0.777 (95% CI: 0.689–0.864, *P*<0.001) for the ADTree model, 0.664 (95% CI: 0.560–0.768, *P* = 0.0033) for the MSKCC nomogram and 0.620 (95% CI: 0.509–0.731, *P* = 0.0032) for the Russells Hall Hospital scoring system.

**Appendix A**

**Appendix B**

**Appendix B (continued)**

**Appendix C**

**Appendix D**

**Appendix E**

**Appendix F**
